# Supplementary material for: Inhibition of double‐strand DNA‐sensing cGAS ameliorates brain injury after ischemic stroke
Source: EMBO Mol Med. 2020 Apr 1;12(4):e11002. doi: 10.15252/emmm.201911002 (PMC7136961; doi:10.15252/emmm.201911002)
Supplement: Supplementary file 1 — Appendix [file EMMM-12-e11002-s001.pdf]

Inhibition of double-strand DNA sensing cGAS ameliorates brain injury after ischemic stroke

Qian Li, Yuze Cao, Chun Dang, Bin Han, Ranran Han, Heping Ma, Junwei Hao, and Lihua Wang

Appendix

Contents

Appendix Figure S1

Appendix Figure S2

Appendix Figure S3

Appendix Figure S4

Appendix Figure S5

Appendix Figure S6

Appendix Figure S7

Appendix Figure S8

Appendix Figure S9

Appendix Table S1

Appendix Table S2

Appendix Figure S1

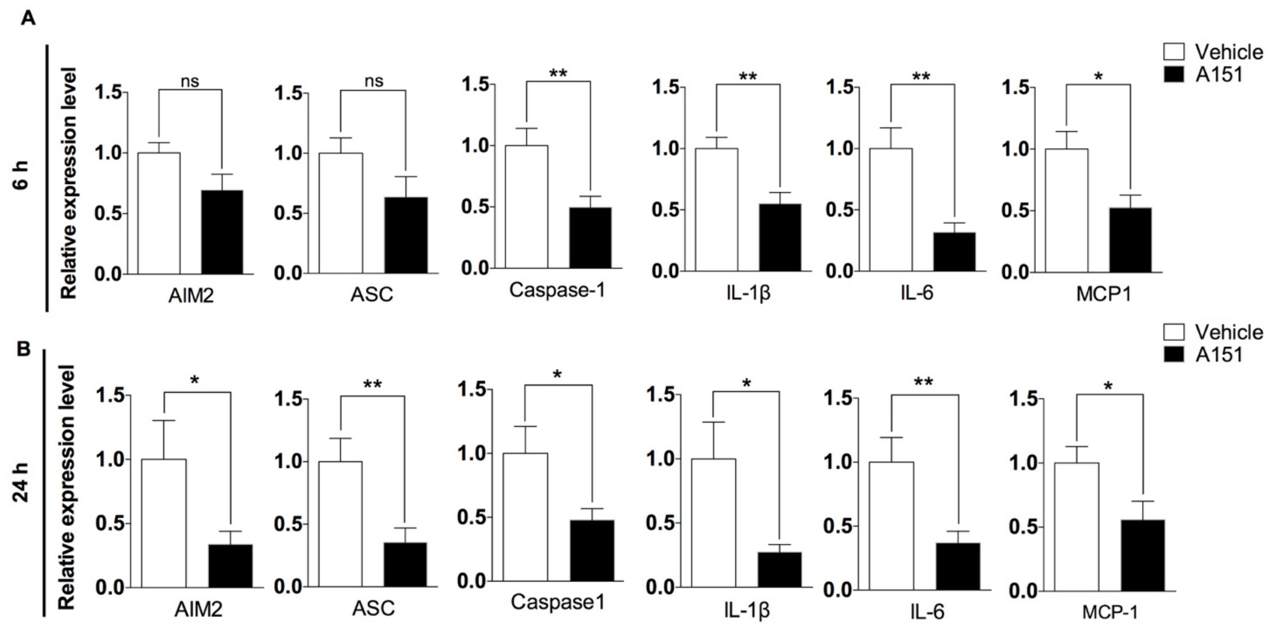

Appendix Figure S1. Administration of A151 dampens the expression of inflammasome-related molecules and inflammatory cytokines at early time points of 6 h and 24 h. The mRNA expression levels of the AIM2 inflammasome components (AIM2/caspase-1/ASC) and levels of IL-1β, and several downstream inflammatory cytokines/chemokines (IL-6, MCP-1) of indicated groups after MCAO at (A) 6 h and (B) 24 h. n = 6 per group. *$P < 0.05$, **$P < 0.01$, two-tailed unpaired Student's t test. Data information: Data are expressed as mean ± SEM. P-values are reported in Appendix Table S2.

Appendix Figure S2

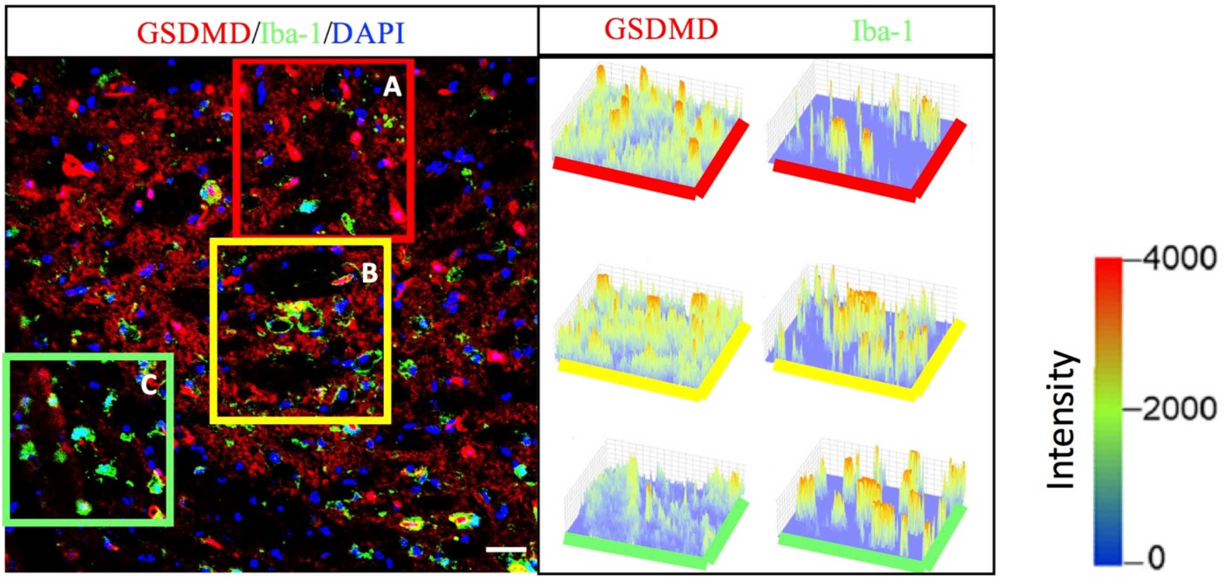

Appendix Figure S2. Microglial pyroptosis stained for Iba-1 and GSDMD in brain of MCAO mice. This representative image illustrates that microglia undergoing pyroptosis (indicated by reference boxes A red, B yellow, and C green). Right panels indicate results of a 2.5D intensity analysis of Iba-1 and GSDMD immunostaining. Scale bar, 20 µm.

Appendix Figure S3

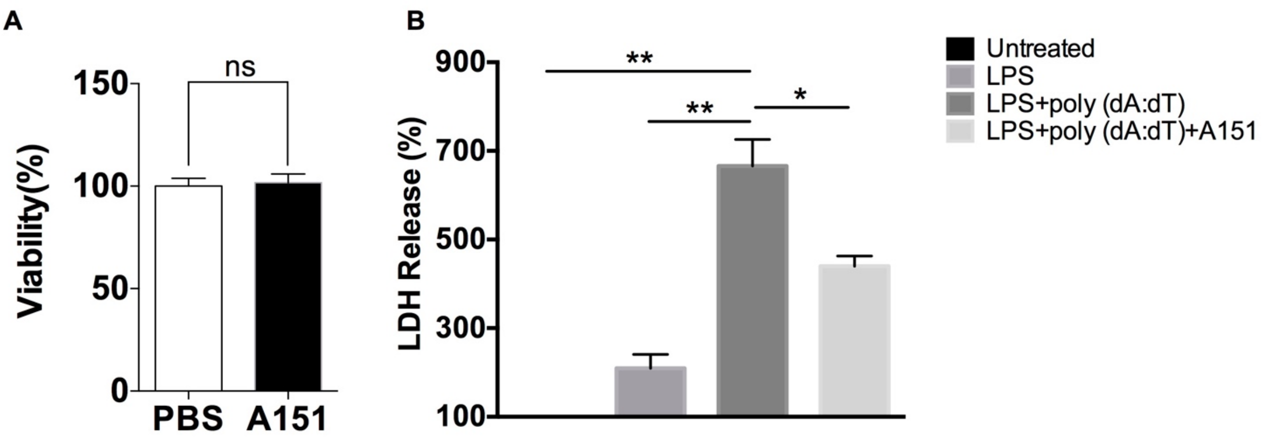

Appendix Figure S3. (A) Cytotoxicity was assessed by MTS assay. P >0.05, two-tailed unpaired Student's t test. (B) Microglial loss was assessed using an LDH activity assay. n = 3 per group. *P < 0.05, **P < 0.01, one-way ANOVA followed by Tukey post hoc test. Data information: Data are expressed as mean ± SEM. P-values are reported in Appendix Table S2.

Appendix Figure S4

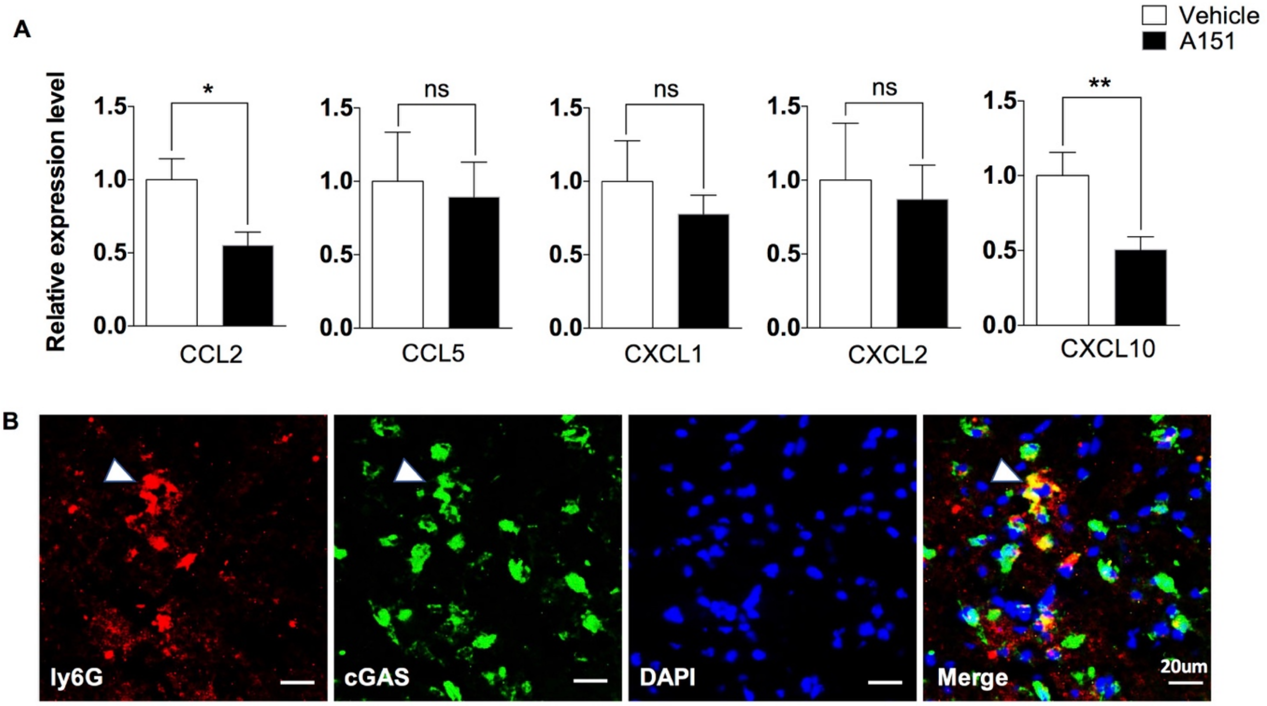

Appendix Figure S4. (A) A151 reduces neutrophil chemoattractants CCL2 and CXCL10. qPCR analysis of chemokines related to the infiltrated neutrophils in vehicle- and A151-treated mice at day 3 after MCAO. n = 6 per group. *P < 0.05, **P < 0.01, two-tailed unpaired Student's t test. (B) Neutrophils co-express cGAS in the penumbra of MCAO mice. Representative immunofluorescence staining for neutrophil marker ly6G (red) and cGAS (green). Data information: Data are expressed as mean ± SEM. P-values are reported in Appendix Table S2.

Appendix Figure S5

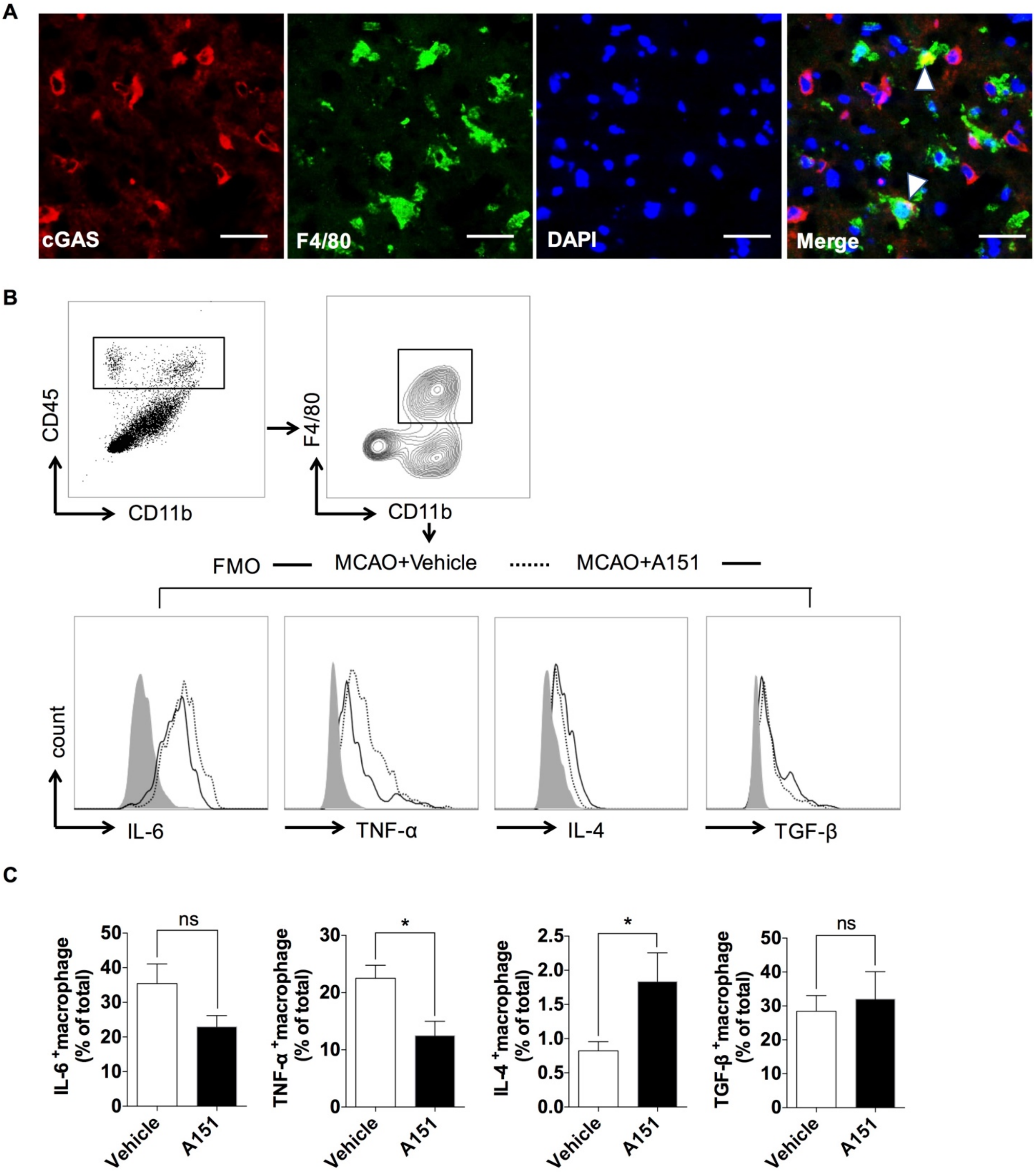

Appendix Figure S5. A151 affects the effect of macrophages. (A) Macrophage marker F4/80 (green) and cGAS (red) co-localize within the same cells (White arrows). (B) Gating strategy for the expression of interlukin-6 (IL-6), IL-4, TNF-α, and TGF-β in macrophages of brains at day 3 after MCAO. (C) Graphs show quantified data. n = 6 per group. *P < 0.05, two-tailed unpaired Student's t test. Data information: Data are expressed as mean ± SEM. P-values are reported in Appendix Table S2.

Appendix Figure S6

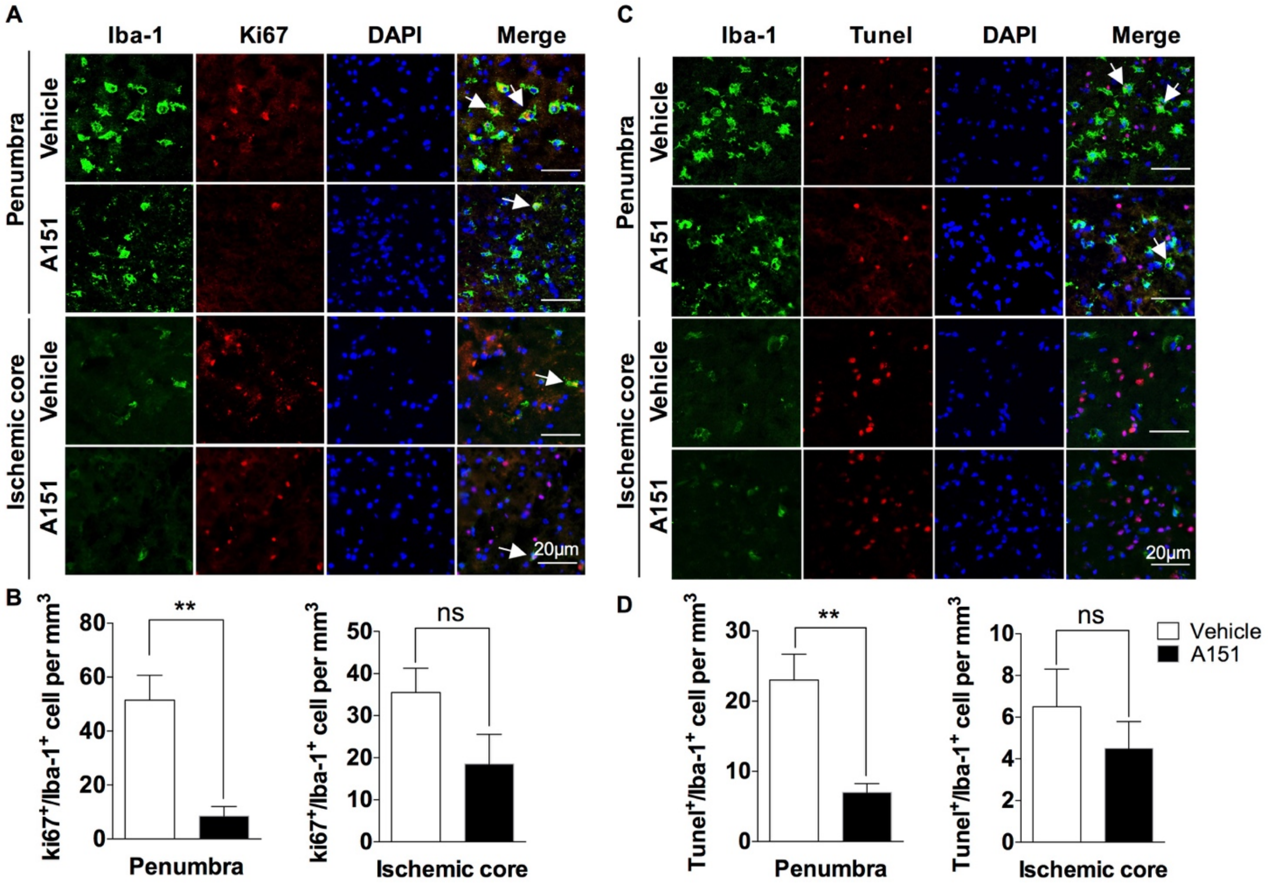

Appendix Figure S6. A151 reduces microglial cell death and proliferation after stroke. (A and C) Representative double immunofluorescence stainings for apoptosis and proliferation of microglia in the penumbra and ischemic core in vehicle - and A151- treated MCAO mice using ki67 (left panel) and TUNEL staining (right panel) at day 3 after MCAO. (B and D) Quantification of ki67/Iba-1 and TUNEL/Iba-1 double-positive cells. n = 6 per group. **P < 0.01, two-tailed unpaired Student's t test. Scale bars, 20 μm. Data information: Data are expressed as mean ± SEM. P-values are reported in Appendix Table S2.

Appendix Figure S7

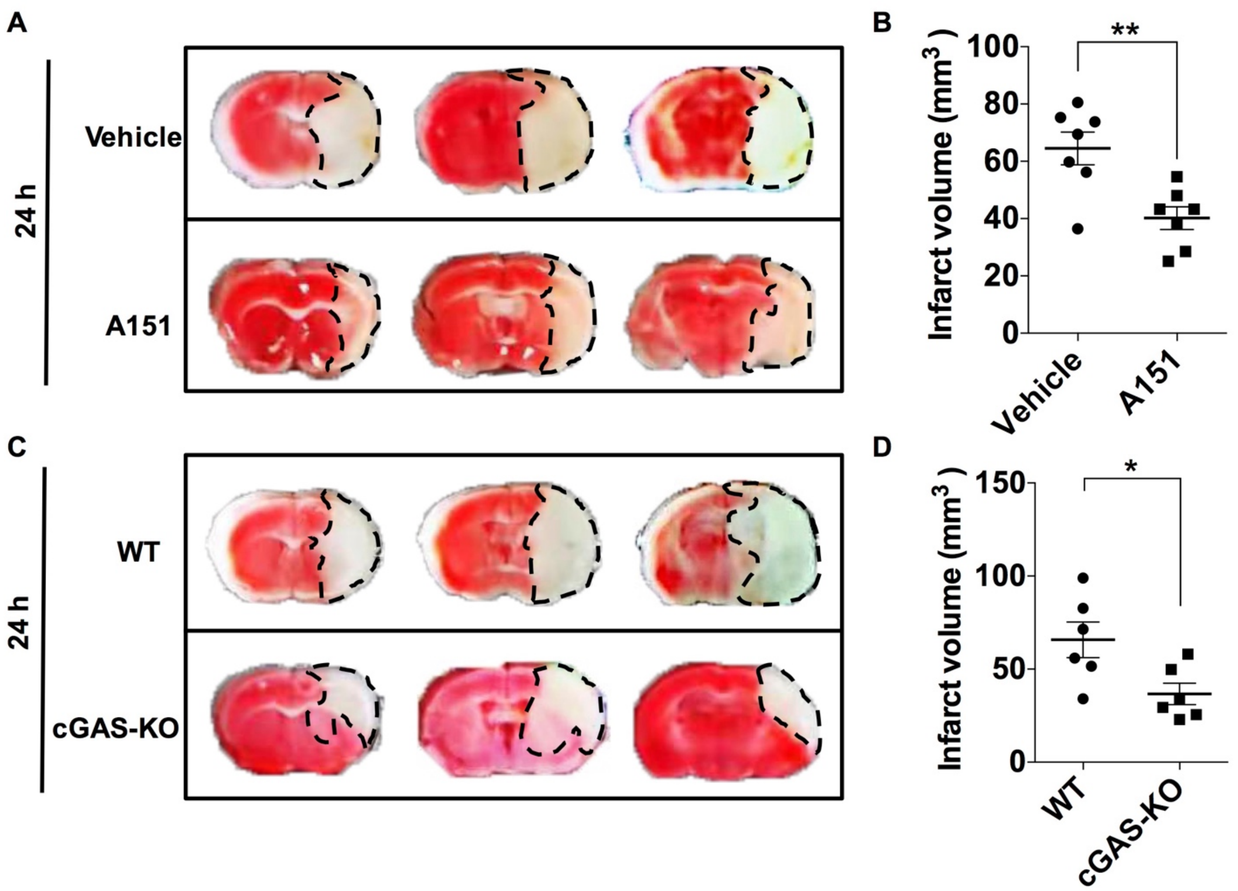

Appendix Figure S7. A151 treatment and microglial cGAS inactivation attenuates ischemic brain injury at 24 h after MCAO. (A) TTC-stained brain slices showing the infarct areas (dark dashed lines) of mice receiving A151 or vehicle at 24 h after reperfusion. The red regions show intact areas; pale regions show infarct areas. (B) Gragh show the infarct volumes in vehicle- and A151-treated MCAO mice. n = 7 per group. **P < 0.01, two-tailed unpaired Student's t test. (C) Representative TTC-stained coronal sections from WT and cGAS-KO mice after 24 h of reperfusion following MCAO. (D) Gragh show the infarct volumes of brain infarct volume. n = 6 per group. *P < 0.05, two-tailed unpaired Student's t test. Data information: Data are expressed as mean ± SEM. P-values are reported in Appendix Table S2.

Appendix Figure S8

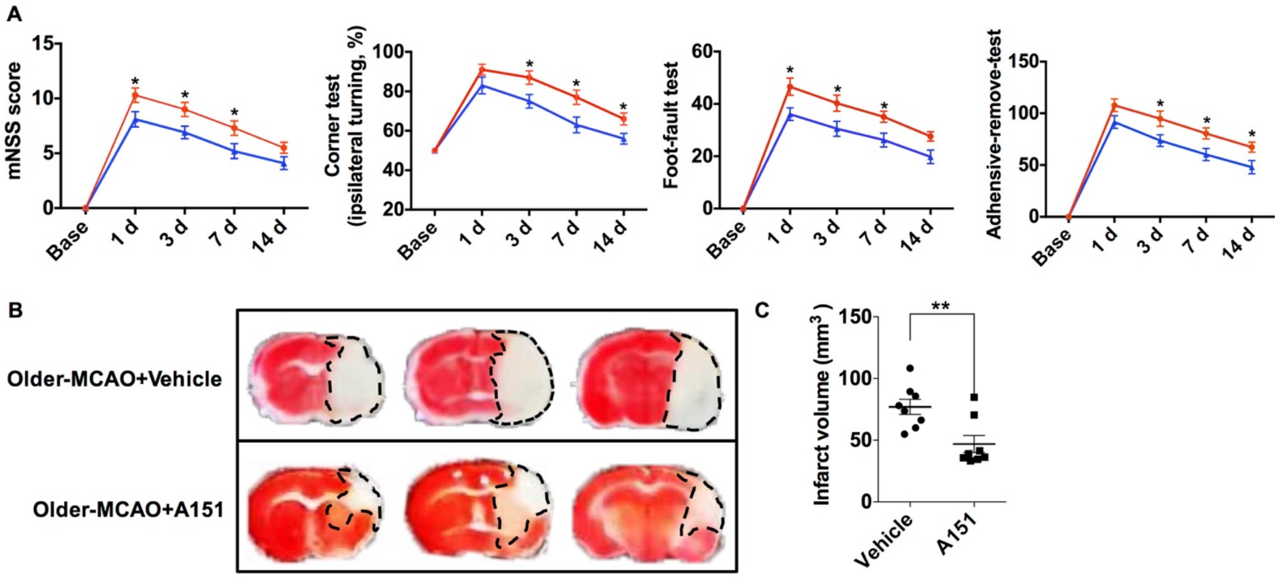

Appendix Figure S8. A151 attenuates brain injury and improves neurological function in older mice after MCAO. (A) Neurological deficits including mNSS score, corner turning test, foot-fault test, and adhesive-removal test were performed at baseline, days 1, 3, 7, and 14 after MCAO. n = 10 per group. *P < 0.05, **P < 0.01, two-way ANOVA with Bonferroni post hoc test. (B) Representative TTC staining images at 24 h after MCAO in vehicle- and A151 treated older mice and (C) quantification of infarct lesions. n = 8 per group. *P < 0.05, two-tailed unpaired Student's t test. Data information: Data are expressed as mean ± SEM. P-values are reported in Appendix Table S2.

Appendix Figure S9

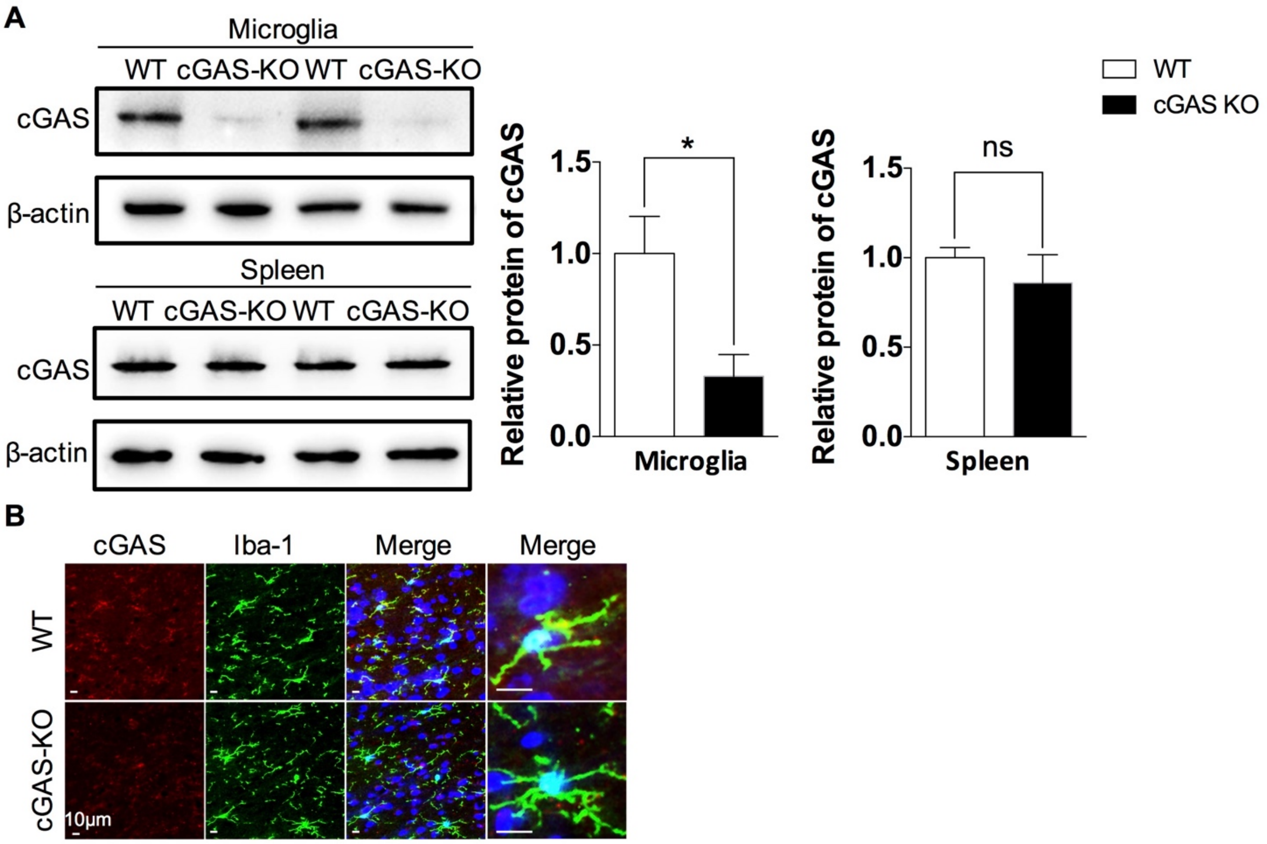

Appendix Figure S9. (A) cGAS expression in microglia and monocytes were assessed by western blot. n = 4 per group. *P < 0.05, two-tailed unpaired Student's t test. (B) Representative double immunofluorescence stainings for cGAS and Iba-1 in WT and cGAS-KO mice. Scale bars, 10 µm. Data information: Data are expressed as mean ± SEM. P-values are reported in Appendix Table S2.

Appendix Table S1. Primers used in the study.

	Primer, 5'–3'	
Gene	Forward	Reverse
CXCL1	CTTGCCTTGACCCTGAAGTCT	AGCAGTCTGTCTTCTTTCTCCGT
CXCL2	AGGGCGGTCAAAAAGTTTGC	CGAGTACGATCCAGGCTTGC
CXCL10	AAGCTATGTGGAGGTGCGAC	AACCCCTTGGGAAGATGGTG
CCL2	CTGCTGTTCACAGTTGCCG	GCACAGACCTCTCTCTTGAGC

	Primer, 5'–3'	
CCL5	GTGCTCCAATCTTGCAGTAG	GGATTACTGAGTGGCATCCCC
IL-6	AGCCAGAGTCCTTCAGAGAG	AGGAGAGCATTGGAAATTGGGG
MCP-1	ACGCTTCTGGGCCTGTTGTT	CCTGCTGCTGGTGATTCTCT

Appendix Table S2. List of exact P-values

**Fig 1D**	**Individual P Value**
contralateral vs. lpsilateral cGAS	0.000530374
contralateral vs. lpsilateral STING	0.000917775
**Fig 1F**	**Individual P Value**
Sham vs. MCAO cGAS	< 0.0001
**Figure 2A**	**Individual P Value**
cGAS	
Sham+Vehicle vs. MCAO+Vehicle	0.0025
Sham+Vehicle vs. MCAO+A151	0.9539
MCAO+Vehicle vs. MCAO+A151	0.0058
STING	
Sham+Vehicle vs. MCAO+Vehicle	0.0006
Sham+Vehicle vs. MCAO+A151	0.6844
MCAO+Vehicle vs. MCAO+A151	0.0078
**Fig 2C**	**Individual P Value**
cGAS	
Sham+Vehicle vs. MCAO+Vehicle	0.0006
Sham+Vehicle vs. MCAO+A151	0.1527
MCAO+Vehicle vs. MCAO+A151	0.0295
STING	
Sham+Vehicle vs. MCAO+Vehicle	0.0021
Sham+Vehicle vs. MCAO+A151	0.3808
MCAO+Vehicle vs. MCAO+A151	0.0322
NF-kB	
Sham+Vehicle vs. MCAO+Vehicle	0.0006
Sham+Vehicle vs. MCAO+A151	0.1046
MCAO+Vehicle vs. MCAO+A151	0.0426
**Figure 2D**	**Individual P Value**
AIM2	
Sham+Vehicle vs. MCAO+Vehicle	0.0041
Sham+Vehicle vs. MCAO+A151	0.8827

MCAO+Vehicle vs. MCAO+A151	0.0154
ASC	
Sham+Vehicle vs. MCAO+Vehicle	0.0003
Sham+Vehicle vs. MCAO+A151	0.2252
MCAO+Vehicle vs. MCAO+A151	0.0101
Caspase-1	
Sham+Vehicle vs. MCAO+Vehicle	0.0047
Sham+Vehicle vs. MCAO+A151	0.8712
MCAO+Vehicle vs. MCAO+A151	0.0187
GSDMD	
Sham+Vehicle vs. MCAO+Vehicle	0.0314
Sham+Vehicle vs. MCAO+A151	0.9835
MCAO+Vehicle vs. MCAO+A151	0.0477
IL-1β	
Sham+Vehicle vs. MCAO+Vehicle	0.0001
Sham+Vehicle vs. MCAO+A151	0.1323
MCAO+Vehicle vs. MCAO+A151	0.039
IL-18	
Sham+Vehicle vs. MCAO+Vehicle	0.0011
Sham+Vehicle vs. MCAO+A151	0.3912
MCAO+Vehicle vs. MCAO+A151	0.0415
**Fig 2F**	**Individual P Value**
AIM2	
Sham+Vehicle vs. MCAO+Vehicle	0.0252
Sham+Vehicle vs. MCAO+A151	0.9971
MCAO+Vehicle vs. MCAO+A151	0.0291
ASC	
Sham+Vehicle vs. MCAO+Vehicle	0.005
Sham+Vehicle vs. MCAO+A151	0.5568
MCAO+Vehicle vs. MCAO+A151	0.0404
caspase-1	
Sham+Vehicle vs. MCAO+Vehicle	0.0028
Sham+Vehicle vs. MCAO+A151	0.6665
MCAO+Vehicle vs. MCAO+A151	0.0158
IL-1β	
Sham+Vehicle vs. MCAO+Vehicle	0.0056
Sham+Vehicle vs. MCAO+A151	0.8255
MCAO+Vehicle vs. MCAO+A151	0.0182
GSDMD	
Sham+Vehicle vs. MCAO+Vehicle	0.0053
Sham+Vehicle vs. MCAO+A151	0.7918
MCAO+Vehicle vs. MCAO+A151	0.0198
**Figure 3B**	**Individual P Value**

IL-1β	
Sham+Vehicle vs. MCAO+Vehicle	< 0.0001
Sham+Vehicle vs. MCAO+A151	0.1155
MCAO+Vehicle vs. MCAO+A151	0.0089
Caspase-1	
Sham+Vehicle vs. MCAO+Vehicle	< 0.0001
Sham+Vehicle vs. MCAO+A151	< 0.0001
MCAO+Vehicle vs. MCAO+A151	0.0003
GSDMD	
Sham+Vehicle vs. MCAO+Vehicle	< 0.0001
Sham+Vehicle vs. MCAO+A151	0.0107
MCAO+Vehicle vs. MCAO+A151	< 0.0001
**Figure 3E**	**Individual P Value**
GSDMD	
Sham+Vehicle vs. MCAO+Vehicle	< 0.0001
Sham+Vehicle vs. MCAO+A151	0.6282
MCAO+Vehicle vs. MCAO+A151	< 0.0001
Caspase-1	
Sham+Vehicle vs. MCAO+Vehicle	0.0001
Sham+Vehicle vs. MCAO+A151	0.9008
MCAO+Vehicle vs. MCAO+A151	0.0005
IL-1β	
Sham+Vehicle vs. MCAO+Vehicle	< 0.0001
Sham+Vehicle vs. MCAO+A151	0.6244
MCAO+Vehicle vs. MCAO+A151	0.0008
**Figure 4B**	**Individual P Value**
cGAS	
Untreated vs. LPS	0.0006
Untreated vs. LPS+poly (dA:dT)	< 0.0001
Untreated vs. LPS+poly (dA:dT)+A151	0.0005
LPS vs. LPS+poly (dA:dT)	0.0008
LPS vs. LPS+poly (dA:dT)+A151	0.9977
LPS+poly (dA:dT) vs. LPS+poly (dA:dT)+A151	0.001
STING	
Untreated vs. LPS	0.1355
Untreated vs. LPS+poly (dA:dT)	0.0001
Untreated vs. LPS+poly (dA:dT)+A151	0.0063
LPS vs. LPS+poly (dA:dT)	0.0012
LPS vs. LPS+poly (dA:dT)+A151	0.1881
LPS+poly (dA:dT) vs. LPS+poly (dA:dT)+A151	0.0191
NF-kB	
Untreated vs. LPS	0.0249
Untreated vs. LPS+poly (dA:dT)	0.0004
Untreated vs. LPS+poly (dA:dT)+A151	0.1758

LPS vs. LPS+poly (dA:dT)	0.0338
LPS vs. LPS+poly (dA:dT)+A151	0.5336
LPS+poly (dA:dT) vs. LPS+poly (dA:dT)+A151	0.0054
ASC	
Untreated vs. LPS	0.0289
Untreated vs. LPS+poly (dA:dT)	< 0.0001
Untreated vs. LPS+poly (dA:dT)+A151	0.0367
LPS vs. LPS+poly (dA:dT)	0.0034
LPS vs. LPS+poly (dA:dT)+A151	0.998
LPS+poly (dA:dT) vs. LPS+poly (dA:dT)+A151	0.0028
IL-1β (FL)	
Untreated vs. LPS	0.2067
Untreated vs. LPS+poly (dA:dT)	0.0002
Untreated vs. LPS+poly (dA:dT)+A151	0.0178
LPS vs. LPS+poly (dA:dT)	0.002
LPS vs. LPS+poly (dA:dT)+A151	0.3537
LPS+poly (dA:dT) vs. LPS+poly (dA:dT)+A151	0.0176
Caspase-1 (FL)	
Untreated vs. LPS	0.0038
Untreated vs. LPS+poly (dA:dT)	0.0002
Untreated vs. LPS+poly (dA:dT)+A151	0.0007
LPS vs. LPS+poly (dA:dT)	0.0813
LPS vs. LPS+poly (dA:dT)+A151	0.4814
LPS+poly (dA:dT) vs. LPS+poly (dA:dT)+A151	0.5545
GSDMD	
Untreated vs. LPS	0.0701
Untreated vs. LPS+poly (dA:dT)	0.009
Untreated vs. LPS+poly (dA:dT)+A151	0.8475
LPS vs. LPS+poly (dA:dT)	0.4779
LPS vs. LPS+poly (dA:dT)+A151	0.2166
LPS+poly (dA:dT) vs. LPS+poly (dA:dT)+A151	0.0266
**Figure 4D**	**Individual P Value**
caspase-1	
Untreated vs. LPS	0.0566
Untreated vs. LPS+poly (dA:dT)	0.0002
Untreated vs. LPS+poly (dA:dT)+A151	0.0203
LPS vs. LPS+poly (dA:dT)	0.0068
LPS vs. LPS+poly (dA:dT)+A151	0.8789
LPS+poly (dA:dT) vs. LPS+poly (dA:dT)+A151	0.0177
IL-1β	
Untreated vs. LPS	0.5143
Untreated vs. LPS+poly (dA:dT)	0.0044
Untreated vs. LPS+poly (dA:dT)+A151	0.5949
LPS vs. LPS+poly (dA:dT)	0.0283

LPS vs. LPS+poly (dA:dT)+A151	0.9988
LPS+poly (dA:dT) vs. LPS+poly (dA:dT)+A151	0.0231
**Fig 5B**	**Individual P Value**
Vehicle vs. A151	
$CD45^{high}CD11b^{+}ly6G^{+}$	0.0056367
$CD45^{high}CD11b^{+}F4/80$	0.102738
$CD45^{high}CD3^{+}CD4^{+}$	0.864721
$CD45^{high}CD3^{+}CD8^{+}$	0.897487
$CD45^{high}CD3^{-}NK1.1^{+}$	0.983523
$CD45^{high}CD3^{-}CD19^{+}$	0.830728
**Fig 5C**	**Individual P Value**
Vehicle vs. A151	
$CD11b^{+}ly6G^{+}$	0.00834513
$CD11b^{+}F4/80$	0.181893
$CD3^{+}CD4^{+}$	0.931188
$CD3^{+}CD8^{+}$	0.37043
$CD3^{-}NK1.1^{+}$	0.898708
$CD3^{-}CD19^{+}$	0.473554
**Fig 5D**	**Individual P Value**
Vehicle vs. A151	
$CD11b^{+}CD45^{high}$	0.0214
**Fig 5E**	**Individual P Value**
Vehicle vs. A151	
IL-4	0.6613
TGF-β	0.002
TNF-α	0.0088
IL-6	0.0301
**Fig 5G**	**Individual P Value**
Vehicle vs. A151	
$ly6G^{+}$ cells/mm^{3}	0.0051
**Fig 5I**	**Individual P Value**
Vehicle vs. A51	
$Iba\text{-}1^{+}$ cells/mm3	0.0025
**Figure 6B**	**Individual P Value**
Vehicle vs. A151	
mNSS	
1 d	< 0.0001
3 d	< 0.0001
7 d	< 0.0001
14 d	0.0025
Corner test	
1 d	0.0003

3 d	< 0.0001
7 d	0.0065
14 d	0.0432
Foot fault test	
1 d	< 0.0001
3 d	0.0003
7 d	0.0098
14 d	0.2169
Adhensive test	
1 d	< 0.0001
3 d	< 0.0001
7 d	< 0.0001
14 d	0.0473
**Figure 6D**	**Individual P Value**
Infact volume	
Sham vs. Vehicle	< 0.0001
Sham vs. A151	< 0.0001
Vehicle vs. A151	0.0159
edema volume	
Vehicle vs. A151	0.0059
**Figure 6F**	**Individual P Value**
Vehicle vs. A151	0.0004
**Figure 6H**	**Individual P Value**
Vehicle vs. A151	0.0031
**Figure 7G**	**Individual P Value**
WT MCAO+Vehicle vs. cGAS KO MCAO+Vehicle	0.0113
WT MCAO+Vehicle vs. cGAS KO MCAO+A151	0.0055
cGAS KO MCAO+Vehicle vs. cGAS KO MCAO+A151	> 0.9999
**Figure 7H**	**Individual P Value**
day 1	
WT MCAO+Vehicle vs. cGAS KO MCAO+Vehicle	< 0.0001
WT MCAO+Vehicle vs. cGAS KO MCAO+A151	< 0.0001
cGAS KO MCAO+Vehicle vs. cGAS KO MCAO+A151	> 0.9999
day 3	
WT MCAO+Vehicle vs. cGAS KO MCAO+Vehicle	0.0001
WT MCAO+Vehicle vs. cGAS KO MCAO+A151	< 0.0001
cGAS KO MCAO+Vehicle vs. cGAS KO MCAO+A151	> 0.9999
**Appendix Figure S1A**	**Individual P Value**
Vehicle vs. A151	
AIM2	0.0586
ASC	0.0954
Caspase-1	0.0053

IL-1β	0.0014
IL-6	0.0008
MCP-1	0.0105
**Appendix Figure S1B**	**Individual P Value**
Vehicle vs. A151	
AIM2	0.047
ASC	0.0058
Caspase-1	0.0295
IL-1β	0.0178
IL-6	0.0056
MCP-1	0.028
**Appendix Figure S3A**	**Individual P Value**
PBS vs. A151	0.5982
**Appendix Figure S3B**	**Individual P Value**
Untreated vs. LPS	0.9999
Untreated vs. LPS+poly (dA:dT)	0.0001
Untreated vs. LPS+poly (dA:dT)+A151	0.0097
LPS vs. LPS+poly (dA:dT)	0.0003
LPS vs. LPS+poly (dA:dT)+A151	0.1281
LPS+poly (dA:dT) vs. LPS+poly (dA:dT)+A151	0.0228
**Appendix Figure S4A**	**Individual P Value**
Vehicle vs. A151	
CCL2	0.0127
CCL5	0.7927
CXCL1	0.4693
CXCL2	0.7748
CXCL10	0.0088
**Appendix Figure S5C**	**Individual P Value**
Vehicle vs. A151	
IL-6	0.0843
TNF-a	0.0141
IL-4	0.0445
TGF-β	0.7087
**Appendix Figure S6B**	**Individual P Value**
Penumbra. ki67$^+$Iba-1$^+$ cell/mm^3	0.0014
Ischemic core. ki67$^+$Iba-1$^+$ cell/mm^3	0.0921
**Appendix Figure S6D**	**Individual P Value**
Penumbra. Tunel$^+$Iba-1$^+$ cell/mm^3	0.0021
Ischemic core. Tunel$^+$Iba-1$^+$cell/mm^3	0.3875
**Appendix Figure S7B**	**Individual P Value**
Vehicle vs. A151	0.0048
**Appendix Figure S7D**	**Individual P Value**
Vehicle vs. A151	0.0259

**Appendix Figure S8A**	**Individual P Value**
Vehicle vs. A151	
mNSS	
1 d	0.0391
3 d	0.0221
7 d	0.0295
14 d	0.7042
Corner test	
1 d	0.2719
3 d	0.0105
7 d	0.0219
14 d	0.0438
Foot fault test	
1 d	0.0111
3 d	0.0209
7 d	0.045
14 d	0.1075
Adhensive test	
1 d	0.1728
3 d	0.0317
7 d	0.0329
14 d	0.0494
**Appendix Figure S8C**	**Individual P Value**
Vehicle vs. A151	0.0057
**Appendix Figure S9A**	**Individual P Value**
WT vs. cGAS-KO	
Microglia	0.029
Spleen	0.4313
